# The many facets of neuropathic arthropathy

**DOI:** 10.1259/bjro.20180039

**Published:** 2019-07-29

**Authors:** R L S Chan, C H Chan, H F Chan, N Y Pan

**Affiliations:** Princess Margaret Hospital, Department of Radiology, Hong Kong,

## Abstract

Neuropathic arthropathy, also known as Charcot arthropathy, refers to progressive and occasionally rapid joint destruction that results from underlying disorders of the nervous system. This pictorial essay aims to illustrate various radiologic findings in neuropathic arthropathy using various examples in the upper and lower limbs and in the spine. Pearls for radiologic diagnosis, clinical differential considerations and possible complications are discussed individually for each joint. MR imaging techniques for differentiating infection and neuropathic arthropathy are explained with examples. Management issues are outlined.

## Introduction

### History

Neuropathic osteoarthropathy was first described by Mitchell in 1831. It was subsequently described by Charcot in 1868, associating it with tabes dorsalis and establishing a causative relationship between neurologic entities and arthropathies. In 1936, Jordan first noted the relationship between diabetic neuropathy and arthropathy, which is currently the commonest aetiology of Charcot joint.^1^

### Pathophysiology

There are many theories regarding the pathophysiology of neuropathic arthropathy, which mainly describe a combination of autonomic, sensory or motor neural dysfunction together with superimposed minor trauma, hyperaemia and increased osteoclast activity, ultimately leading to joint destruction.^1^

In the early stages of the disease, there is cartilaginous fragmentation leading to *d*istention of the joint, with normal or increased bone *d*ensity. Subsequent *d*estruction of subchondral bone leads to intraarticular *d*ebris. In later stages, the joint is *d*eformed due to progressive osseous destruction, with possible *d*islocation due to ligamentous laxity from injury of periarticular stabilizing soft tissue structures. This description of the pathophysiologic process encloses the 6 “D"s that characterise neuropathic arthropathy.^2^

### Clinical presentation

Acutely, the joint is usually swollen with mild erythema and increased temperature. Pain may be present. Infection is an important differential consideration in the acute stage. In the chronic stage, there is less erythema, but swelling usually persists and the joint is structurally deformed.

### Radiologic appearance

Radiographically, neuropathic arthropathy can be classified into hypertrophic, atrophic and mixed forms.^3^ The hypertrophic form features debris, fragmentation, sclerosis and exuberant osteophytes as predominant findings. It mainly involves the spine and large joints in the lower limb, especially the knees. The appearance may mimic severe osteoarthritis. The atrophic form features bone resorption as the predominant feature. It mainly involves non-weight bearing locations and the forefoot. The appearance may mimic septic arthritis especially at the shoulder. The mixed form is common, and is characterized by a combination of hypertrophic and atrophic changes.

### Diagnosis

Diagnosis is predominantly based on typical appearances on radiographs, or by monitoring progressive changes on serial radiographs. Aspiration of joint fluid is often performed at initial diagnosis to exclude septic arthritis. CT is generally not required. MRI of the cervical spine is helpful to evaluate for syringomyelia in cases of upper limb neuropathic arthropathy.

**Figure 1.**
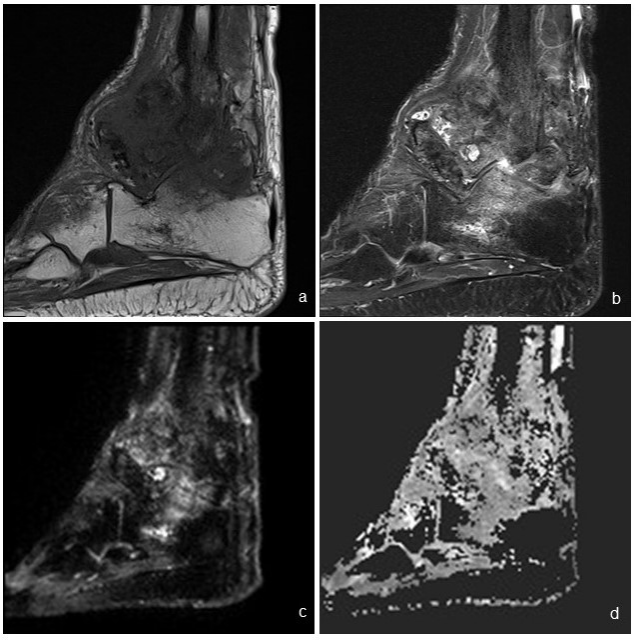
Selected sagittal MR images of the right ankle in a syphilitic patient with known neuropathic neuroarthropathy. T1 (a) and T2 (b) images show predominantly subarticular marrow edema at the calcaneum. Such distribution points toward marrow edema due to neuropathic arthropathy rather than osteomyelitis. DWI b700 (c) and ADC (d) images show no restricted diffusion at the region of marrow oedema, which favours the absence of osteomyelitis. In the latter, restricted diffusion is expected due to presence of inflammatory infiltrates.

Osteomyelitis the most common and important differential. Both infection and neuropathic joint may show rim-enhancing synovial fluid collections thus posing diagnostic difficulty. MRI can be helpful in differentiating the two entities, by distribution of the bone marrow edema, and looking for specific findings pointing towards infection such as subcutaneous abscesses, sinus tracts and skin ulcers (Figure 1). Diffusion-weighted imaging has also recently been suggested as an aid for differentiating neuropathic arthropathy from infection (Figure 1).^4^

Patient demographics and the location of the involved joint may suggest different aetiologies of neuropathic arthropathy. Each aetiology has a different predilection for individual joints (Table 1) and is associated with slightly different radiographic features. A detailed discussion is given below.

**Table 1.** Common sites of involvement of various aetiologies of neuropathic arthropathy

**Disease**	**Site of Involvement**
**Diabetes and other causes of peripheral neuropathy** (such as alcohol, leprosy and Charcot-Marie-Tooth disease)	Foot and ankle, (wrist less common)
**Syringomyelia**	Shoulder, elbow, wrist
**Spinal cord injury**	Spine, knee
**Congenital insensitivity to pain**	Multiple joints
**Tabes dorsalis**	Knee, hip

## Upper extremity

### Shoulder

Neuropathic arthropathy is uncommon in the upper extremity, where the shoulder is the most susceptible target of involvement. Presentation is usually painless swelling due to fluid distension of the glenohumeral joint and subacromial-subdeltoid bursa.

Radiologically, the atrophic type predominates. In the most severe cases, the entire humeral head and neck can be resorbed, mimicking prior surgical resection. Specific radiological findings such as superomedial flattening of humeral head, periarticular soft tissue calcification and glenoid sclerosis may be present.^5^

**Figure 2.**
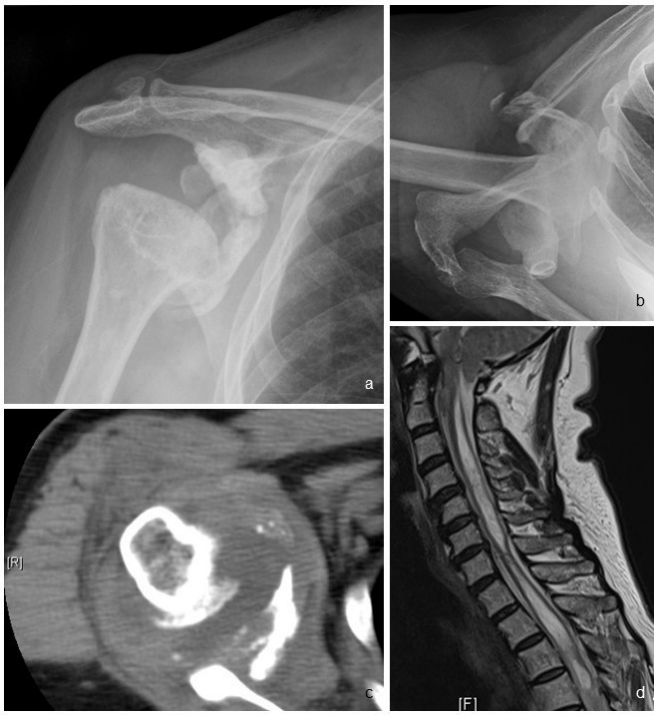
(a) Frontal right shoulder radiograph of a 52-year-old female patient shows deformity of the right humeral head and glenoid with preserved bone density. The humeral head is inferiorly subluxed. (b) Axial view shows anterior dislocation of humeral head. (c) Axial CT slice of the right shoulder shows glenohumeral joint fluid with debris within. Microbiological culture of aspirated joint fluid was negative. (d) With a radiological diagnosis of neuropathic right shoulder, MRI of cervical spine was performed which shows a long-segment syringomyelia extending through cervical and thoracic levels.

Syringomyelia is implicated in up to 80% of cases (Figure 2). Syringomyelia is a cystic collection in the spinal cord around the central canal. It causes progressive motor neuropathy leading to claw hands, neuropathic arthropathy, and sensory neuropathy predominantly affecting temperature and pain sensation.

Other less common causes include diabetes, Arnold-Chiari malformation, post-traumatic syringomyelia and cervical spondylosis. Management is by protective immobilization of the shoulder with a sling and restricting activity. Surgical options are usually limited due to a lack of remaining bone stock.

### Elbow

**Figure 3.**
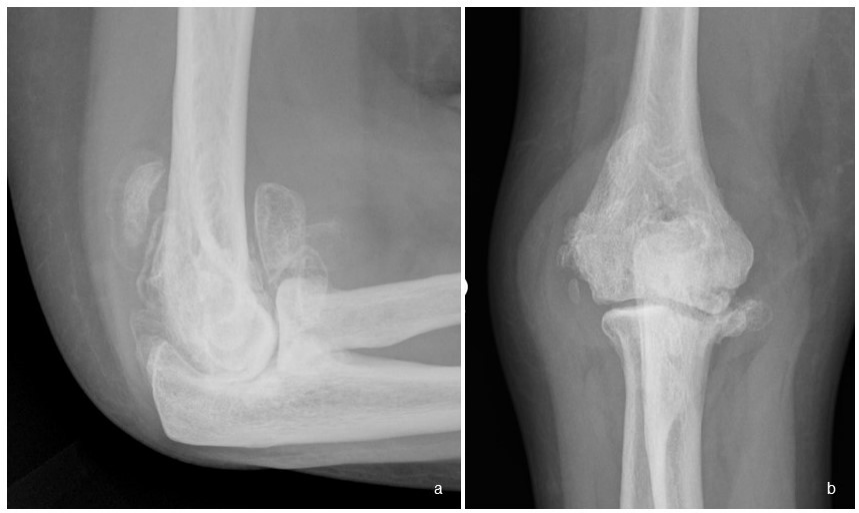
(a, b). Frontal and lateral radiographs of the right elbow of a patient with syringomyelia (same patient as Figure 2) show significant debris within the elbow in keeping with a more commonly hypertrophic appearance as compared to neuropathic arthropathy involving the shoulder. Also typical to neuropathic arthropathy is increased density, joint distension and mild deformity of the proximal ulna.

**Figure 4.**
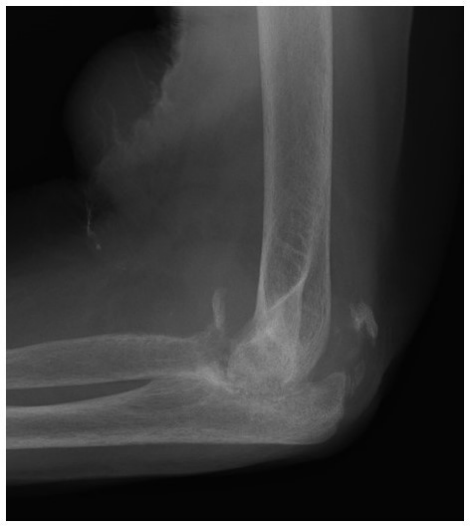
Lateral radiograph of the left elbow of a 74-year-old female patient with diabetes shows destruction and resorption of proximal radius and ulna, and debris located away from the joint, likely within the large amount of joint fluid. Findings are all compatible with neuropathic arthropathy.

**Figure 5.**
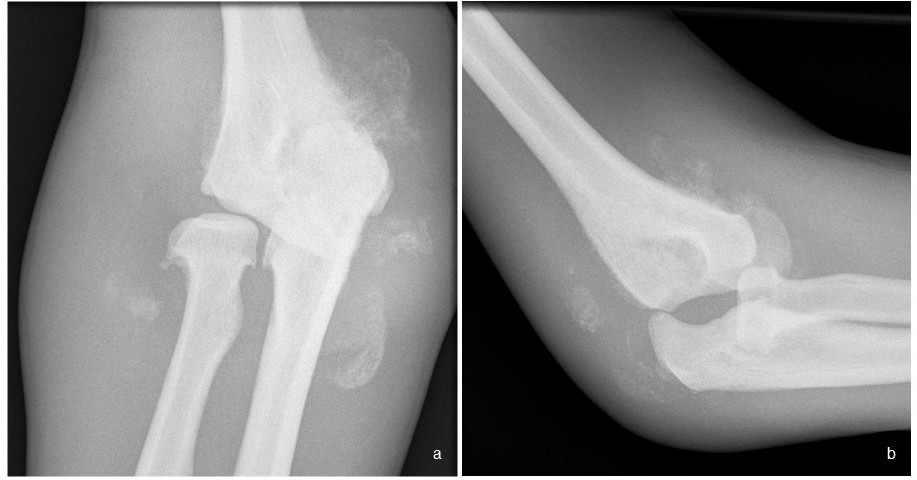
(a, b). Frontal and lateral right elbow radiographs of a 34-year-old male patient with congenital insensitivity to pain show deformed distal humerus and proximal ulna with preserved bone density. Extensive debris within large amount of joint fluid is typical of neuropathic elbow. Lateral view confirms dislocated elbow joint, which is related to the concomitant destruction of periarticular soft tissues and ligaments.

Elbow joint involvement is uncommon. Swelling with instability or dislocation is a common presentation. Radiologically, the mixed hypertrophic and atrophic type is usually seen and is associated with significant debris.^6^ As with the shoulder, neuropathic arthropathy of the elbow joint is most commonly caused by syringomyelia (Figure 3). Other described aetiologies include diabetes (Figure 4), syphilis, and congenital insensitivity to pain (Figure 5).

Compression of the ulnar nerve and posterior interosseous nerve are known complications of neuropathic elbow which are not uncommon.

Functional bracing is usually performed, allowing flexion and extension while aiming to neutralize varus and valgus stresses.

### Wrist

Neuropathic arthropathy of the wrist is rare. Thus, relevant specific clinical presentations and radiological features have not yet been well described. Case reports have been published, citing diabetes mellitus, leprosy, congenital indifference to pain, syringomyelia and syphilis as aetiologies.

## Spine

Traumatic spinal cord injury is currently the commonest aetiology, most frequently affecting the thoracolumbar spine. Mobile segments caudad to the stabilized segment of the spine are at risk, with physiotherapy being the source of repeated movement in paraplegic patients.^7^ Spinal involvement was previously classically described in tabes dorsalis, which is now almost a historical entity due to introduction of vigorous antibiotic treatment in early stages of the disease.^8^

**Figure 6.**
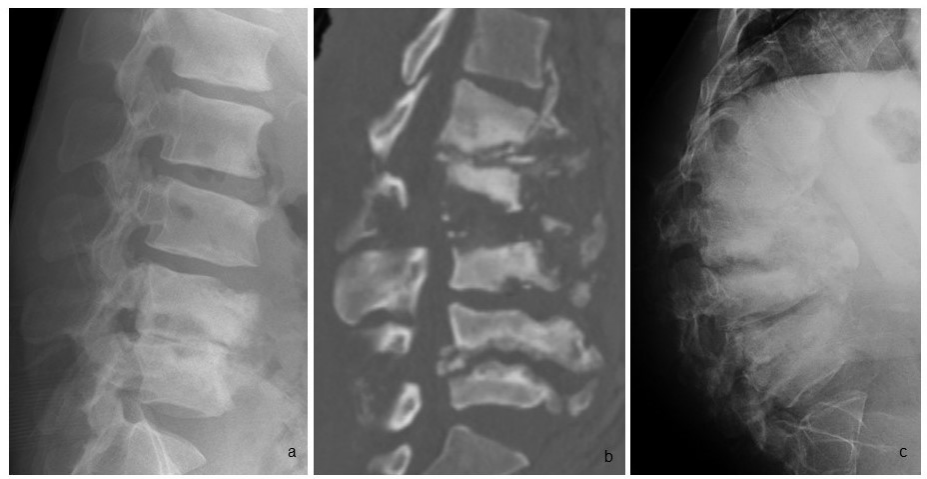
Serial lumbar spine imaging of a 34-year-old patient with congenital insensitivity to pain. Elbow radiographs of the same patient are shown in Figure 5. (a) Lateral lumbar spinal radiograph shows severe loss of the L4/5 disc space with adjacent end-plate erosion. (b) CT lumbar spine performed 2 years later shows extensive lumbar vertebral body fragmentation and disc degeneration but preserved bone density. Abundant osseous debris and facet joint involvement (not shown) are typical of neuropathic osteoarthropathy and uncommon in infective spondylodiscitis. (c) Lateral lumbar radiograph taken after another 4 months shows further deterioration with resultant lumbar kyphotic deformity.

Other described causes include congenital insensitivity to pain and diabetes mellitus. Congenital insensitivity to pain is a group of hereditary sensory and autonomic neuropathies causing a lack of deep pain sensation, leading to progressive painless destruction of multiple large joints. In addition, osteomyelitis, dislocations and fractures may also occur in affected patients (Figure 6).^9^

**Figure 7.**
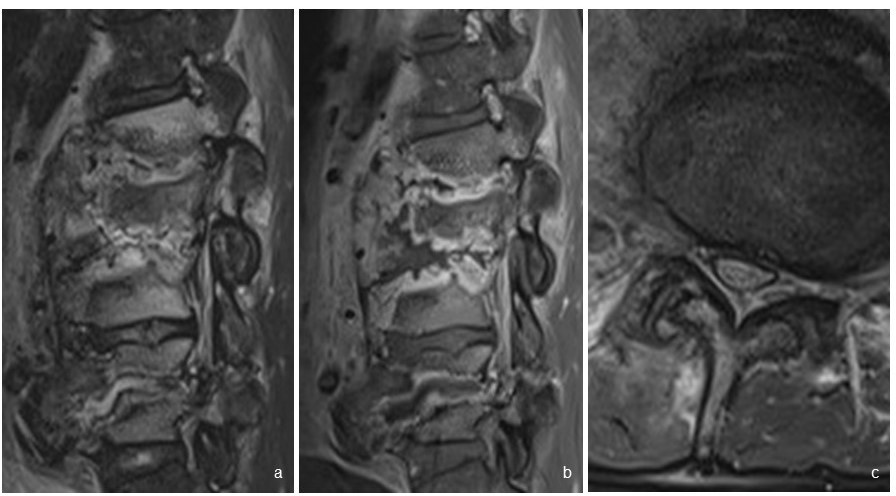
(a, b, c). (a) Sagittal turbo spin-echo fat-suppressed *T*_2_ weighted image of a 34-year-old patient with congenital insensitivity to pain (same patient as Figure 6) shows high signal intensity in L1 and L3 vertebral bodies. (b) Sagittal turbo spin-echo gadolinium enhanced *T*_1_ weighted image shows rim-enhancement of L1/2, L2/3 and L4/5 discs, typical of neurospondyloarthropathy. (c) Erosion of right facet joint at L4/5, typical of the 3-column involvement in spinal neuroarthropathy. The MRI features may superficially mimic multifocal infective spondyloarthritis, although the latter does not usually simultaneously involve the facet joints.

Imaging may help to distinguish between spondylodiscitis and neuropathic arthropathy. Neuropathic spinal arthropathy usually shows significant osseous debris and joint disorganization, and involves the facet joints in addition to the end plates (Figure 7, MRI in same patient as in Figure 6).^10^

Management of neuropathic spine is initially conservative. If there is significant instability, circumferential arthrodesis may be performed to secure both the anterior and posterior columns.

## Lower extremity

### Foot and ankle

The foot and ankle is the most commonly affected region in neuropathic arthropathy. Presenting features include warmth, swelling, erythema and deformity. Special to the foot and ankle region, involvement of any joint affects biomechanics of other joints due to alteration of weight-bearing force transmission and increased weight-bearing stresses, leading to a severe destructive arthropathy of multiple joints as the disease progresses.

**Figure 8.**
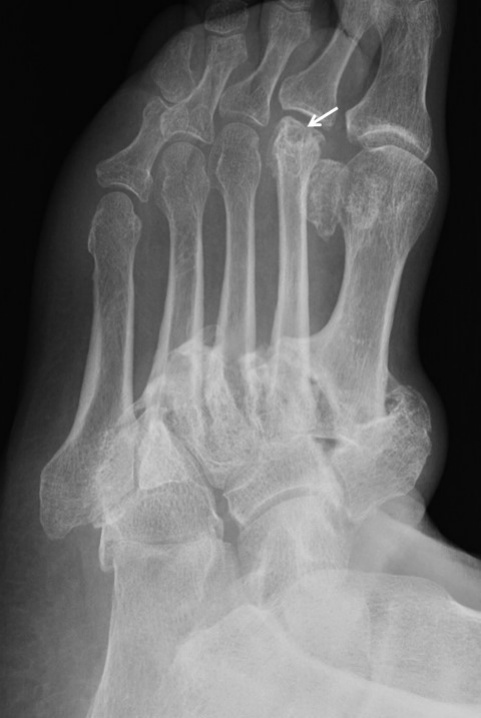
Brodsky Type one neuropathic arthropathy in a 67-year-old male patient with diabetic peripheral neuropathy. Oblique radiograph of his left foot shows homolateral dislocation of Lisfranc joint leading to deformity with exuberant osteophyte formation. There was no significant history of prior trauma. Also of note is the flattened head of second metatarsal (white arrow), with a similar appearance to Freiberg infarction - a well known location for neuropathic fracture.

**Figure 9.**
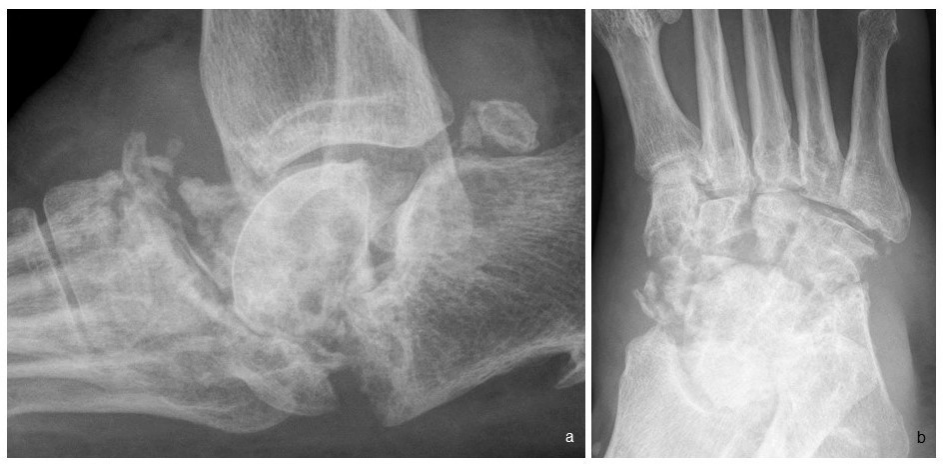
(a, b). Brodsky Type two neuropathic arthropathy in a 53-year-old female patient with Charcot Marie Tooth disease. Lateral and Oblique radiographs of her right foot show involvement of the intertarsal and subtalar joints and significant resorption of tarsal bones suggestive of predominant atrophic appearance. Associated significant swelling around the joint is likely due to joint fluid. Flattening of the plantar arch is a classic feature of neuropathic foot.

**Figure 10.**
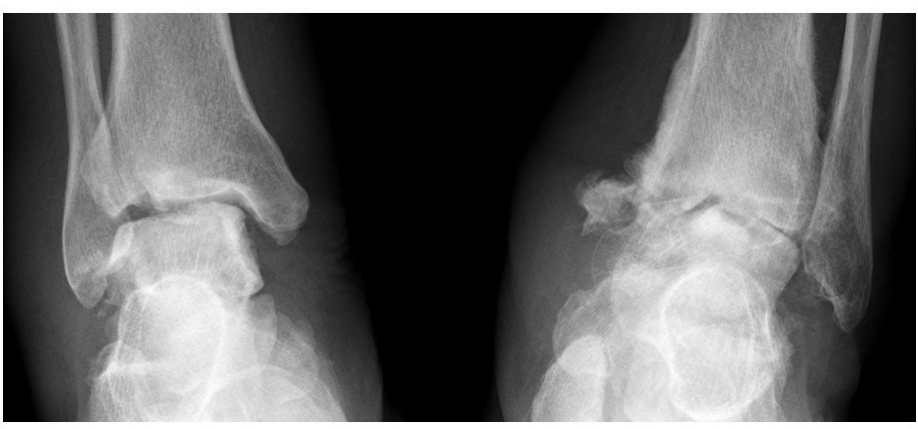
Brodsky Type three neuropathic arthropathy in a 65-year-old male patient with alcoholic neuropathy. Frontal radiograph of both ankles shows destruction of both tibiotalar joints with preserved bone density. Significant debris and joint swelling present at left mortise joint. Prognosis for this patient is worse compared to patients with forefoot or midfoot involvement.

**Figure 11.**
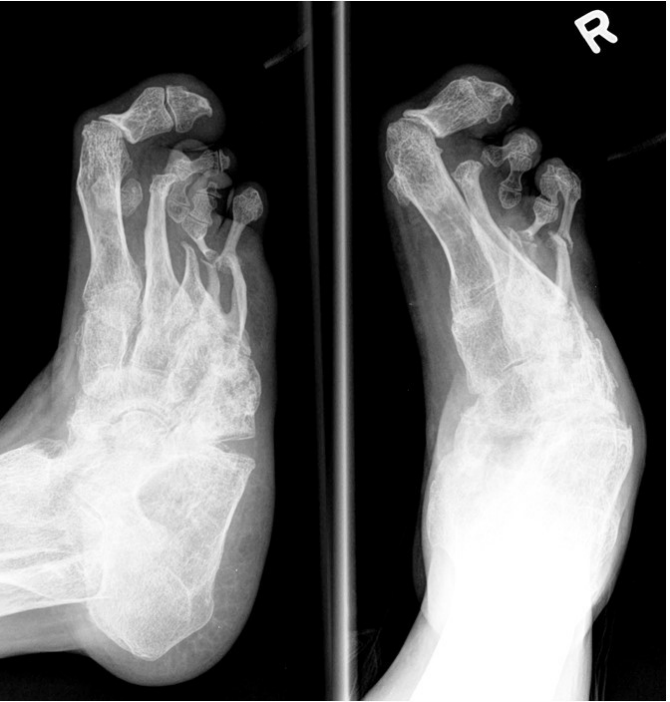
Brodsky Type five neuropathic arthropathy with forefoot involvement in a 69-year-old female patient with leprosy. Dorsal-plantar and Oblique radiographs of her right foot show licked candy stick appearance of the second to fifth metatarsals with predominant atrophic pattern, typical in patients with peripheral neuropathy due to leprosy. Some of the phalanges have been completely resorbed.

The Brodsky Classification describes the different radiologic patterns of joint involvement within the foot and ankle. Type one involves the Lisfranc and naviculocuneiform joints, and has the highest incidence (Figure 8, in a patient with diabetic neuropathy). Type two involves the talonavicular, calcaneocuboid and subtalar joints (Figure 9, in a patient with Charcot-Marie-Tooth disease), while Type three involves the tibiotalar joint (Figure 10, in a patient with alcoholic neuropathy). Type five involves the forefoot, and is the least common (Figure 11, in a patient with leprosy). Type four describes involvement of multiple regions.^11^

Diabetes causes a predominantly sensory neuropathy. It is the most frequent aetiology of neuropathic foot and ankle, usually involving only the foot. It is unilateral in 75% of cases. The mixed hypertrophic and atrophic type is the most common form. Other radiological findings include vascular calcifications, infection, and neuropathic fractures. Other sites of involvement are less common (Figure 8).^12^

Charcot-Marie-Tooth disease is a heterogeneous group of hereditary mixed neuropathies with a predominant motor component. It usually affects the lower limb, especially the foot. Associated features include pes cavus, hammer toes, scoliosis, muscle atrophy and hypertrophic nerves (Figure 9).

Chronic alcoholism frequently co-exists with diabetes mellitus and thiamine deficiency, but can also independently cause peripheral neuropathy. Features of pure alcoholic neuroarthropathy have been described in case reports, and are similar to those of diabetic neuropathic arthropathy (Figure 10).^13^

Leprosy is a neurocutaneous infection caused by *Mycobacterium leprae* that results in a mixed sensory and motor neuropathy. Bone and joint changes at the interphalangeal and metatarsophalangeal joints are more frequently seen in leprosy, and the atrophic type is also more common, as compared to other aetiologies. Concomitant findings include deformities due to motor neuropathy, ulceration, infection, and neuropathic fractures (Figure 11).

Other less commonly implicated causes include congenital insensitivity to pain and myelomeningocele. Prognosis is worse for proximal (hindfoot or ankle) neuropathic arthropathy, with a higher incidence of complications such as deformity, ulceration and osteomyelitis. There is also a higher chance of amputation. Management is initially conservative with foot care such as casting or bracing to prevent ulceration and infection. In severe cases, arthrodesis is performed to re-establish a normal weight-bearing axis and restore a plantigrade foot.^11^

### Knee

**Figure 12.**
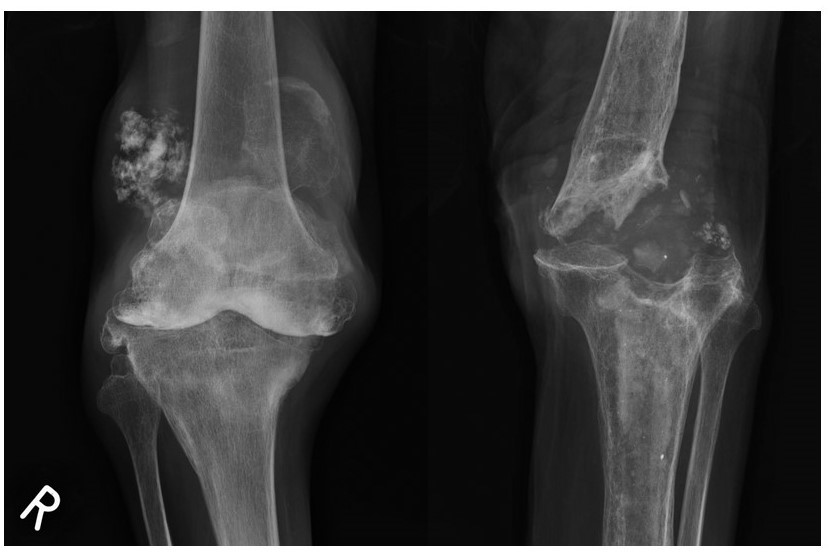
Frontal knee radiographs of a 82-year-old female patient with history of spinal cord injury at young age with paraparesis show features of bilateral knee neuropathic arthropathy. There is bone resorption of left femoral condyles and lateral tibial plateau with fragmentation. The right tibiofemoral joint is subluxed with incidental finding of osteochondromatosis. Generalized osteopenia, atypical in neuropathic arthropathy, is not confined to the knee joint and likely related to long term disuse.

**Figure 13.**
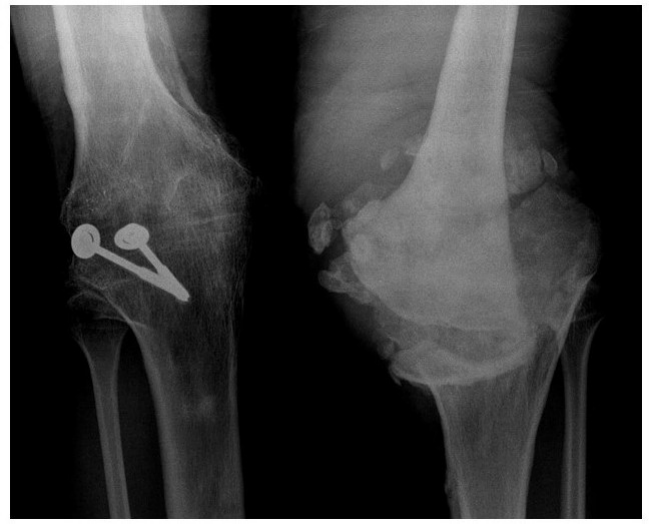
Frontal radiograph of both knees of a 34-year-old patient with known congenital insensitivity of pain (Figures 5 and 6 show elbow and spine involvement in the same patient). Left tibiofemoral joint is dislocated and deformed. Bone density is preserved but there is bone resorption at the left femoral condyles and tibial plateau. Significant swelling is present, with debris within the joint. Findings are typical of left knee neuropathic arthropathy with both hypertrophic and atrophic features. Right tibiofemoral joint was surgically fused, due to history of neuropathic right knee with repeated dislocations despite bracing.

Knee joint involvement is uncommon, and is predominantly described in case reports. The knee is a major weight-bearing joint, and neuropathic involvement can lead to significant clinical instability. Other common presenting symptoms include joint swelling and crepitus. Radiologically, hypertrophic features predominate but are sometimes mixed with atrophic components. Traumatic spinal cord injury can lead to neuropathic arthropathy of the spine and lower limb joints including the knees. (Figure 12) Other described causes include congenital insensitivity to pain (Figure 13), diabetes mellitus, and tabes dorsalis.

Management is initially conservative with protective bracing. In severe cases, arthrodesis may be performed during the quiescent phase of the disease, with fair rates of success.^14^

### Hip

**Figure 14.**
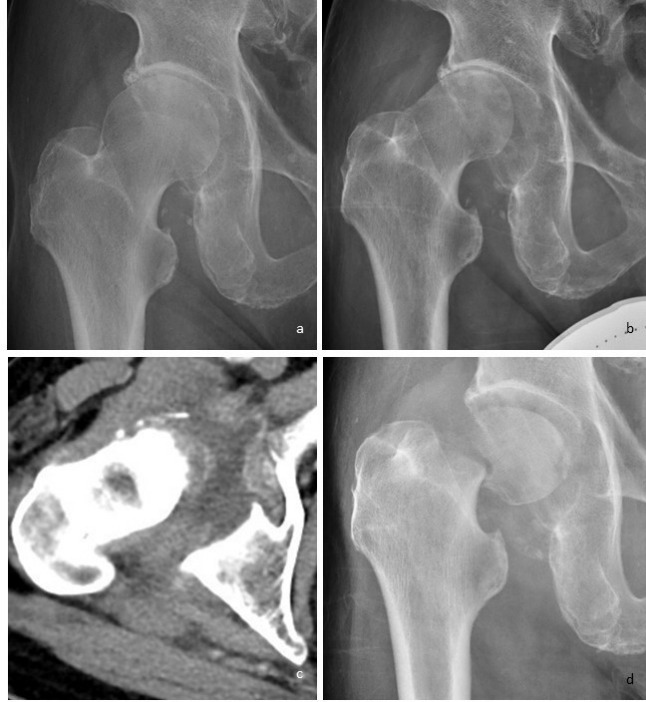
Serial imaging of the right hip in a 80-year-old male patient subsequently diagnosed to have idiopathic right neuropathic hip. (a) First frontal right hip radiograph shows no significant abnormality. (b) Second radiograph performed 1 month apart shows deformed right femoral head with loss of superior hip joint space but preserved bone density. (c) Selected axial image from the right hip CT scan performed 1 week later shows increased hip joint effusion with osseous debris, typical of hypertrophic neuropathic hip. Culture of aspirated joint fluid yielded no growth. (d) Subsequent radiograph after 2 weeks shows fracture of femoral neck with associated fragmentation and intra articular debris formation, despite no history of trauma. There is also associated bone resorption at the fracture site. Rapidly destructive osteoarthropathy is an important imaging differential diagnosis which has similar appearance, as it is also associated with joint space narrowing. However, it typically shows more significant acetabular involvement, and usually does not cause intraarticular fractures and debris formation.

**Figure 15.**
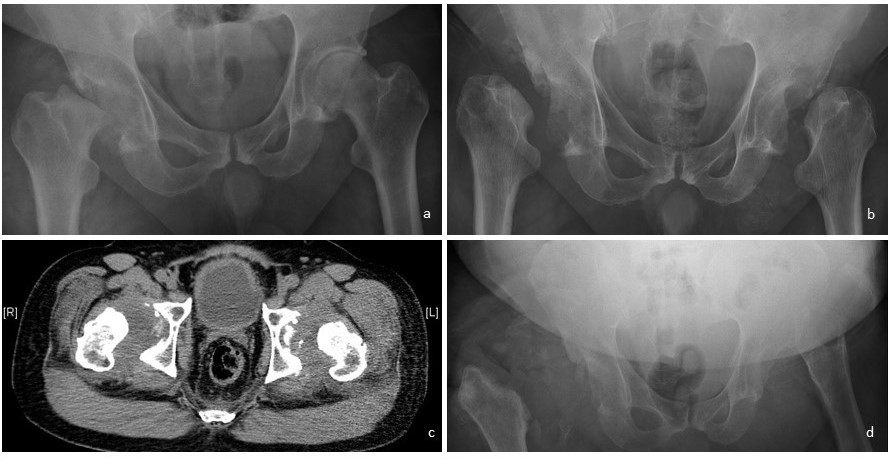
(a) Frontal pelvic radiograph of a 52-year-old male patient who presented with painless limping shows resorption of right femoral head. (b) Subsequent radiograph 2 months apart shows resorption of left femoral head as well. (c) Selected axial CT image of bilateral hip joints shows bilateral joint effusions with minimal debris. Relative sparing of the acetabula despite marked resorption of femoral head is common in neuropathic arthropathy. Repeated joint aspiration is negative for microbial culture. Radiological diagnosis of neuropathic joint is thus made. Patient’s VDRL blood test is positive, but lumbar puncture is not performed thus syphilis status in cerebral spinal fluid cannot not be determined. (d) Another pelvic radiograph performed 4 months apart shows complete resorption of bilateral femoral head and neck with dislocation.

Neuropathic arthropathy of the hip is rare. Protective factors have been postulated, including relatively abundant nerve supply of the hip relative to peripheral joints, and less wear and tear due to better protection by adjacent soft tissue and muscles. Previously, tabes dorsalis was the most common cause of neuropathic arthropathy of the hip, but nowadays it is often idiopathic. The two predominant radiological patterns of involvement described are^15^ – (1) Fracture of the femoral head or neck due to minimal trauma (Figure 14, in an idiopathic case), which is usually managed by internal fixation; or (2) extensive joint destruction with resorption of significant portion of the femoral head and neck (Figure 15, in a patient with suspected latent syphilis), which is usually managed supportively.

Apart from septic arthritis, rapidly destructive osteoarthritis is another common differential of neuropathic hip. Although rapidly destructive osteoarthritis may show some overlap features with neuropathic arthropathy (mainly destruction of the femoral head), most features are distinct from one another^16^ (Table 2).

**Table 2.** Features for differentiating neuropathic arthropathy and rapidly destructive osteoarthritis of the hip

**Neuropathic arthropathy of the hip**	**Rapidly destructive osteoarthritis of the hip**
Male > Female	Usually Female
Painless, or minimal pain disproportionate to degree of arthropathy	Pain is present invariably
Unilateral or bilateral	Majority are unilateral
Intraarticular debris is common in the hypertrophic type	Intraarticular debris is not a characteristic feature
Intraarticular fracture may be present	Intraarticular fracture is not a characteristic feature
Disproportionate lysis of femoral head, with relative sparing of the acetabulum may be present	Both acetabular and femoral side are usually affected to similar degree
Joint space narrowing and subchondral sclerosis are not characteristic features	Joint space narrowing and subchondral sclerosis are present in most cases

Management differs for the two different radiological subtypes of neuropathic hip. Internal fixation is performed for cases with femoral neck fracture. On the other hand, management is initially conservative for cases showing the resorptive pattern, as surgery is usually unsuccessful due to limited remaining bone stock. Arthrodesis, bone graft or total hip arthroplasty may be considered for advanced cases.

## Conclusion

Neuropathic arthropathy is an uncommon condition with significant morbidity. It is important for radiologists to be familiar with the various radiologic features of this disease. In this pictorial review, examples of various affected joints and a variety of aetiologies of neuropathic arthropathy have been reviewed. Assessment of the pattern of small-joint involvement in the foot and ankle is helpful, as it can point to different aetiologies and carries prognostic and management implications. If upper limb joints are involved, MR imaging of the cervical spine is indicated to look for syringomyelia. MR imaging can also help differentiate between infection and neuropathic arthropathy. Pearls for radiologic diagnosis, clinical differential considerations, possible complications and recent management issues have been outlined and discussed individually for each joint.
